# Single-port robotic-assisted wet colostomy after total pelvic exenteration: a feasibility video report

**DOI:** 10.3389/fonc.2025.1698531

**Published:** 2026-01-12

**Authors:** Carlo Ronsini, Maria Cristina Solazzo, Mariano Catello Di Donna, Giuseppe Cucinella, Cono Scaffa, Vito Chiantera

**Affiliations:** Unit of Gynecologic Oncology, National Cancer Institute, Istituto di Ricovero e Cura a Carattere Scientifico (IRCCS), Fondazione “G. Pascale”, Naples, Italy

**Keywords:** endometrial cancer recurrence, minimally invasive reconstruction, single-port robotic surgery, total pelvic exenteration, wet colostomy

## Abstract

**Background:**

Total pelvic exenteration (TPE) is a radical procedure involving the en bloc removal of pelvic organs. Among reconstructive strategies, wet colostomy—a single conduit for both urinary and fecal diversion—offers advantages such as reduced operative time and lower risk of ureteral anastomotic complications.

**Objective:**

The aim of this study was to describe a case of wet colostomy performed entirely using a single-port (SP) robotic-assisted approach, demonstrating the feasibility of this minimally invasive technique.

**Methods:**

A case report study was conducted at the Gynecologic Oncology Unit of the Istituto Nazionale Tumori “Fondazione Pascale” in Italy. A pelvic recurrence of endometrial cancer was treated using the Da Vinci SP^®^ robotic system. The voice-commented video article details both the TPE and reconstructive procedures. Primary outcomes included technical description, operative time, hospital stay, and blood transfusions.

**Results:**

In January 2025, a 68-year-old woman with pelvic recurrence of endometrial carcinoma underwent robotic-assisted TPE with wet colostomy. The procedure lasted 320 min, with an estimated blood loss of 50 cc, requiring no transfusion. The patient was discharged on postoperative day 4 without complications within the first 30 days.

**Conclusion:**

This is the first reported case of TPE with robotic SP-assisted wet colostomy. The video illustrates the surgical strategy and highlights the feasibility of this innovative technique.

**Synopsis:**

This report describes the technique of single-port robotic-assisted wet colostomy following total pelvic exenteration for recurrent endometrial cancer, providing a step-by-step overview of the surgical approach.

## 1 Introduction

Total pelvic exenteration (TPE) is a radical surgical procedure performed in patients with locally advanced or recurrent pelvic malignancies (1). In recurrent patients, it involves the en bloc removal of the bladder, rectum, distal ureters, vagina, and surrounding tissues. Initially described by Brunschwig in 1948 (2), this highly morbid intervention remains one of the most extensive oncologic surgeries, with an estimated morbidity rate of 50%–80% and a 5-year survival rate of approximately 30%–50% in selected patients (3). Despite its complexity, TPE remains a potentially curative option for cases with isolated pelvic recurrence in the absence of distant metastases (4).

The choice of urinary and fecal diversion following exenteration is a critical factor influencing postoperative morbidity and quality of life (5). Among reconstructive strategies, wet colostomy, a technique where a single conduit serves for both urinary and fecal diversion, has emerged as a simplified and effective alternative (6, 7). This method is associated with reduced operative time, lower rates of ureteral anastomotic strictures, and fewer urinary complications compared to separate urinary diversion techniques such as ileal conduits or continent urinary reservoirs (8, 9). However, wet colostomy is underutilized in current clinical practice despite these benefits.

The introduction of minimally invasive techniques, particularly robotic-assisted surgery, has opened new horizons to pelvic exenteration. It potentially reduces blood loss, perioperative complications, and recovery time (10). While multi-port robotic TPE has been previously reported (11), single-port robotic TPE (SP-RA TPE) has never been described. Single-port surgery offers additional advantages, including reduced surgical trauma, fewer incisions, and improved cosmesis (12).

This report presents the first case of single-port robotic-assisted (SP) TPE with wet colostomy, demonstrating the feasibility of this innovative, minimally invasive approach by step-by-step descriptive video.

## 2 Materials and methods

### 2.1 Patient selection and clinical setting

A 68-year-old woman with a body mass index (BMI) of 26 and a pelvic recurrence of radio-treated endometrial carcinoma underwent single-port robotic-assisted TPE with a wet colostomy at the National Cancer Institute, IRCCS Fondazione “G. Pascale,” Naples, Italy, in January 2025.

The patient had undergone a hysterectomy, bilateral adnexectomy, and systematic pelvic lymphadenectomy in 2020 for stage IIIA endometrial carcinoma, according to FIGO 2009 (13) (pT3N0G2). Following major international guidelines (14), the patient underwent pelvic external beam radiotherapy (47 Gy) and brachytherapy (4 Gy). Negative controls followed until December 2024 where a total body positron emission tomography–computed tomography (PET-CT) showed tracer accumulation at the level of the vaginal dome (28.3 SUV), in the absence of other suspected secondarisms. A multidisciplinary evaluation determined TPE as the best oncologic option.

### 2.2 Surgical planning

To optimize the surgical strategy, the patient underwent an examination under narcosis, which revealed a stenotic vagina that was difficult to explore. The vaginal walls appeared hard-ligneous in consistency as from frank neoplastic infiltration. The vaginal dome appeared unexplorable. Vesico-vaginal septum and vaginal rectum were non-flowing, frankly infiltrated. Suspected infiltration of the anterior wall of the rectum was documented on rectal exploration. Because of the clinical picture, the patient was a candidate for TPE surgery with en bloc removal of pelvic peritoneum, bladder, sigmoid colon and rectum, ureters, total colpectomy, and wet colostomy packing.

### 2.3 Robotic platform

The procedure was performed using the Da Vinci SP^®^ robotic system (Intuitive Surgical, Sunnyvale, CA), which allows multi-instrument articulation through a single access port.

### 2.4 Surgical technique

We have articulated the description of the technique in 20 steps (21 with an optional step), as shown in Video 1 (**Supplementary Materials**).

#### 2.4.1 Step 1. Umbilical access and robotic docking (00:32)

A 5-cm umbilical midline incision was performed using a Hasson open technique, allowing for the placement of a single-port multi-instrument access device. The Da Vinci SP^®^ robotic system was docked, providing 3D high-definition visualization and enhanced instrument maneuverability.

#### 2.4.2 Step 2. Retroperitoneal access and ureteral mobilization (00:52)

The retroperitoneum was accessed using a latero-medial approach. The left paracolic gutter was incised, and the sigmoid colon was mobilized. During this procedure, a pericentimetric nodule was identified on the sigmoid serosa as the most cranial involvement and later confirmed as a cancer recurrence by a frozen section.

#### 2.4.3 Step 3. Prevesical peritoneum incision and paravesical space development (01:45)

The prevesical peritoneum was incised, exposing the umbilical artery. The lateral and medial paravesical spaces were developed, merging with the previously dissected paracolic gutter to improve exposure.

#### 2.4.4 Step 4. Exposure of vascular structures and ureteral dissection (02:10)

Because of prior radiotherapy, the paravesical spaces exhibited significant fibrosis, requiring careful dissection to expose the vascular axes and ureteral course while preventing injuries. Dissection of the urethral tunnel proceeds craniocaudally, with complete opening of Latzko’s lateral pararectal space and Okabayashi’s medial space.

#### 2.4.5 Step 5. Contralateral ureteral mobilization and presacral space development (02:30)

To maintain operative symmetry, the same procedures are repeated contralaterally. The right ureter was fully mobilized, completing bilateral ureteral isolation. Peritoneal dissection proceeds in a manner that maintains approximately 2 cm of margin from macroscopic localizations, circumscribing the prevesical and Douglas peritoneum.

#### 2.4.6 Step 6. Posterior dissection (03:00)

The presacral space was accessed, ensuring preservation of the inferior hypogastric plexus, and allowing mobilization of the intestinal tract to be resected.

#### 2.4.7 Step 7. Ureteral clamping and distal mobilization (03:17)

A clip was placed on the juxtavesical ureter to prevent urine spillage. Approximately 4 cm of the ureter was sacrificed, followed by distal mobilization to the pelvic brim. Closure of the distal section of the ureter results in temporary exclusion of the kidney. However, it prevents urine drainage and its irritative and vision worsening consequences. This arrangement is particularly useful in SP surgery, where the suction system suffers from the lack of ancillary accesses.

#### 2.4.8 Step 8. En bloc isolation of the surgical specimen (03:43)

The tumor-affected pelvic organs (bladder, rectum, vagina, and distal ureters) were circumscribed and isolated for en bloc removal. At this stage, parametrectomy and paracolpectomy of the tracts bordering the tumor infiltration is necessary.

#### 2.4.9 Step 9. Retzius space development and bladder mobilization (04:04)

The Retzius space was developed, allowing for complete mobilization of the bladder. The vesico-vaginal plane was retrogradely dissected, exposing the anterior vaginal wall.

#### 2.4.10 Step 10. Vaginal resection and urethral dissection (04:17)

The anterior vaginal wall was incised, including the urethral segment, to ensure oncologic clearance.

#### 2.4.11 Step 11. Ischiorectal fossae and levator muscle exposure (04:39)

Both ischiorectal fossae were developed, exposing the levator ani muscles. The inferior hypogastric plexus was sacrificed where tumor infiltration was present.

#### 2.4.12 Step 12. Sacral fascia development and medial fusion of compartments (05:06)

A medial plane was created to communicate between the right and left pelvic compartments upon the sacral fascia. This part ends the mobilization of the tumor specimen and should be adjusted based on how craniality of the bowel to be resected is needed.

#### 2.4.13 Step 13. Perineal phase—abdominoperineal resection and colpectomy (out of screen)

Because of tumor extension into the vagina, a perineal phase was performed, including a circular vulvar incision, total colpectomy, and abdominoperineal resection (Miles technique (15)). The perineal plane is then closed again by a nonresorbable staccato stitch suture.

#### 2.4.14 Step 14. Redocking for reconstructive phase (05:46)

Following tumor resection, the robotic system was redocked to begin urinary and fecal reconstruction via wet colostomy.

#### 2.4.15 Step 15. Hemostatic patch on urethral stump (05:50)

A hemostatic patch was placed on the urethral stump to reduce the risk of fistulization, and a bladder catheter was left in place.

#### 2.4.16 Step 16. Ureteral mobilization beyond pelvic brim (06:05)

The ureters were bilaterally mobilized beyond the pelvic brim to prevent kinking caused by positional displacement toward the wet colostomy reservoir.

#### 2.4.17 Step 17. Colonic mobilization (06:24)

The left paracolic incision was extended to the colon flexure, and the end tract of the resected colon was mobilized.

#### 2.4.18 Step 18. Aortic retroperitoneal access (06:42)

Lumbar retroperitoneal access with identification and isolation of the superior hypogastric plexus and inferior mesenteric artery.

#### 2.4.19 Step 19. (Optional) inferior mesenteric artery isolation and ligation (07:07)

Depending on the extent of colonic mobilization, sacrificing the inferior mesenteric artery may be required. Dividing this vessel facilitates the exteriorization of the colonic stump, which serves as the wet colostomy reservoir. However, this step is not mandatory and should be performed only when necessary.

#### 2.4.20 Step 20. Colic and ureteral exteriorization (07:33)

Once the colonic stump and ureters were fully mobilized, the robot was de-docked, and the structures were externalized through the umbilical access. This approach leverages the existing laparotomic incision to complete the reconstructive phase without requiring additional openings. Such a strategy is feasible only due to the extensive minimally invasive mobilization, eliminating the need for further intracorporeal maneuvers.

#### 2.4.21 Step 21. Stoma positioning and finalization (07:59)

The sigmoid colon stump was prepared, and a reservoir was created to ensure isolation from the intestinal transit. A bilateral uretero-colonic anastomosis (wet colostomy) was performed, with mono-J ureteral stents positioned and secured to the skin. The wet colostomy was externalized in the left flank, completing the urinary and fecal diversion.

### 2.5 Variables

The study’s main objective is the video-supported description of TPE and wet colostomy with the SP robotic approach. Operative times, expressed in minutes and calculated from induction of anesthesia, to skin synthesis; blood loss, estimated according to the surgeon’s experience and expressed as cubic centimeters; the tumor residual, expressed as a multimodal variable as 0 (residual absent), <1 (microscopic residual), and >1 macroscopic residual; the days of hospitalization; the need for transfusions both intra- and postoperative; and the postoperative complications in the first 30 days, according to Clavien–Dindo (16) have been collected.

## 3 Results

**Figure 1:**
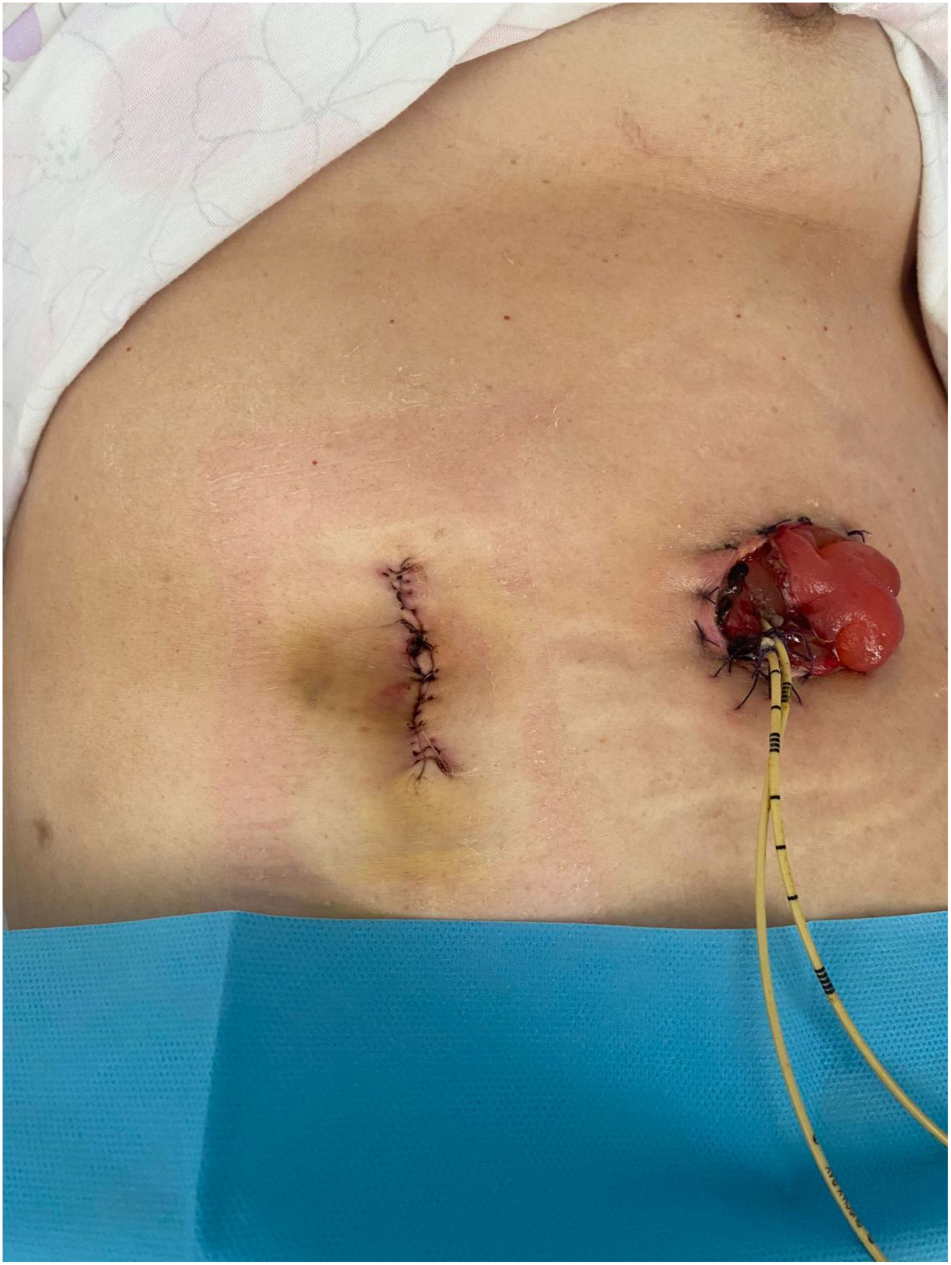
Final view on the day of discharge.

The total operative time was 320 min, with an estimated blood loss of 50 cc. No intraoperative complications occurred, and no blood transfusions were required. A complete (R0) tumor resection was successfully achieved. The patient had an uneventful postoperative course, with a hospital stay of 4 days, and no complications were observed within the first 30 days postoperatively. Cosmetic results are reported in **Figure 1**.

## 4 Discussion

### 4.1 Interpretation of results

This case demonstrates the feasibility of SP-RA TPE with wet colostomy, with low blood loss, early discharge, and no complications. The minimally invasive approach did not compromise oncologic safety. Moreover, the step-by-step description, also supported by the descriptive video, enables standardization of the technique and promotes its dissemination.

### 4.2 Clinical implications

Minimizing abdominal trauma through single-port access may contribute to reduced postoperative pain, fewer wound complications, and a shorter hospital stay. Additionally, a wet colostomy simplifies urinary and fecal diversion, decreasing anastomotic morbidity and facilitating postoperative management. Moreover, the enhanced articulation and maneuverability of instruments in the SP approach improve navigation within the operative field, potentially offering greater surgical precision. While not the primary focus of this manuscript, the SP approach may present a lower learning curve than other minimally invasive techniques, warranting further investigation.

### 4.3 Comparison with literature

Traditional open TPE is associated with high morbidity, extended hospital stays, and complex reconstructions (17). With the advent of minimally invasive surgery, various TPEs with both laparoscopic (18) and robotic (19) approaches have been described, demonstrating advantages in safety, operative timing, and cosmetic outcome (10). However, the reconstructive time is the major limitation of minimally invasive approaches, including the robotic approach (20). Various techniques exist to reconstitute bowel and ureteral continuity. In this scenario, a wet colostomy serves as an innovative alternative to reduce surgical steps and require only one stoma, thereby improving quality of life (21). Multi-port robotic approaches have been reported in both gynecologic, rectal, and urologic pathologies (22–24). In the field of urology, cases of single-port urinary diversion have been described, but never associated with TPE, only with cystectomy (25). However, SP-RA TPE has not been described to date, making this report a pioneering case in robotic exenteration.

### 4.4 Strengths and limitations

The systematic nature of the procedure, with its step-by-step description, is a strength, encouraging the distribution and standardization of this technique. However, the high technical demand and limited availability of single-port robotic platforms restrict widespread adoption. In addition, the technique should be regarded as executable only in experienced hands. This technique involves a learning curve and adaptation of robotic surgical knowledge to the single-port method. Another limitation is related to the distribution of single-port platforms, which are currently only available in certain centers. Therefore, this method cannot be generalized and the technique should be tailored to the specific needs of each clinical case. Future prospective studies should further evaluate this technique in larger patient cohorts. Finally, it should be noted that the choice of a double stoma has a significant impact on patients’ quality of life and is therefore reserved for cases with a poor prognosis, such as the one described above.

## 5 Conclusion

This is the first reported case of SP-RA TPE with a wet colostomy, demonstrating technical feasibility and clinical benefits. Minimally invasive exenteration could represent an evolution in the surgical management of advanced pelvic malignancies, offering lower morbidity and faster recovery.

## Data Availability

The original contributions presented in the study are included in the article/**Supplementary Material**. Further inquiries can be directed to the corresponding author.
